# Comparative Mitogenomics of the Genus *Odontobutis* (Perciformes: Gobioidei: Odontobutidae) Revealed Conserved Gene Rearrangement and High Sequence Variations

**DOI:** 10.3390/ijms161025031

**Published:** 2015-10-20

**Authors:** Zhihong Ma, Xuefen Yang, Miklos Bercsenyi, Junjie Wu, Yongyao Yu, Kaijian Wei, Qixue Fan, Ruibin Yang

**Affiliations:** 1 Key Lab of Freshwater Animal Breeding Certificated by Ministry of Agriculture, College of Fisheries, Huazhong Agricultural University, Wuhan 430070, China; E-Mails: simohorse@163.com (Z.M.); xfy@mail.hzau.edu.cn (X.Y.); wujunjie@webmail.hzau.edu.cn (J.W.); yuyongyao@webmail.hzau.edu.cn (Y.Y.); kjwei@mail.hzau.edu.cn (K.W.); fanqixue@mail.hzau.edu.cn (Q.F.); 2 Freshwater Aquaculture Collaborative Innovation Center of Hubei Province, Wuhan 430070, China; 3 Georgikon Faculty, University of Pannonia, Keszthely 8360, Hungary; E-Mail: miklosbercsenyi77@gmail.com

**Keywords:** mitochondrial genome, codon usage, gene order, control region, tandem repeats, phylogeny

## Abstract

To understand the molecular evolution of mitochondrial genomes (mitogenomes) in the genus *Odontobutis*, the mitogenome of *Odontobutis yaluensis* was sequenced and compared with those of another four *Odontobutis* species. Our results displayed similar mitogenome features among species in genome organization, base composition, codon usage, and gene rearrangement. The identical gene rearrangement of *trnS-trnL-trnH* tRNA cluster observed in mitogenomes of these five closely related freshwater sleepers suggests that this unique gene order is conserved within *Odontobutis*. Additionally, the present gene order and the positions of associated intergenic spacers of these *Odontobutis* mitogenomes indicate that this unusual gene rearrangement results from tandem duplication and random loss of large-scale gene regions. Moreover, these mitogenomes exhibit a high level of sequence variation, mainly due to the differences of corresponding intergenic sequences in gene rearrangement regions and the heterogeneity of tandem repeats in the control regions. Phylogenetic analyses support *Odontobutis* species with shared gene rearrangement forming a monophyletic group, and the interspecific phylogenetic relationships are associated with structural differences among their mitogenomes. The present study contributes to understanding the evolutionary patterns of Odontobutidae species.

## 1. Introduction

The vertebrate mitogenomes are usually small circular molecules (16–18 kb) containing 13 protein-coding genes (PCGs), two rRNA genes (rRNAs), 22 tRNA genes (tRNAs), and a putative control region (CR) [1,2]. Due to its simple structure, constant gene content, rapid evolutionary rate, and maternal inheritance, mtDNA has been extensively used for studying population genetics [3], biogeography [4], and phylogenetics [5,6]. Moreover, it is of great importance to offer genome- and sequence-level information, such as the gene rearrangement [7,8] and the evolutionary patterns of the CR [9].

The CR is usually regarded as the most variable part of the mitogenome in terms of nucleotide substitutions, short insertion/deletion, and variable number of tandem repeats (VNTRs) [10,11]. The tandem repeats in CR of mitogenomes have been documented in a wide range of taxa [10,12,13]. They are usually located in the extended termination-associated sequences (ETAS) domain or conserved sequence blocks (CSBs) domain, which are more variable than the central conserved domain [14]. So far, three main mechanisms have been proposed to explain the formation of the repeated sequences in different regions of the mitochondrial CR [13], including the illegitimate elongation model [15], the improper initiation model [16], and the pause-melting misalignment [17]. These tandem repeats provide a source of length polymorphism and heteroplasmy within individuals and species of particular vertebrate taxa [15,18]. Consequently, comparative analyses of tandem repeats may play a crucial role in studying mitogenome evolution or population dynamics from a population level perspective [19].

As an increasing number of complete mitogenomes of metazoan are sequenced, diverse gene rearrangements have been identified [2,20,21]. For instance, several marsupials and caecilian amphibians have derived rearrangement of *trnW-trnA-trnN-trnC-trnY* to *trnA-trnC-trnW-trnN-trnY* [22,23,24], three crocodilians share the exchange in position of *trnS* and *trnH* [25,26], and 10 parrotfishes possess the shared gene order *trnI-trnM-trnQ* which is different from the typical vertebrate gene order *trnI-trnQ-trnM* [27]. Thus far, there are three models usually applied to explain gene rearrangements in metazoan mitogenomes: Firstly, the recombination model, involving the breaking and rejoining of DNA strands [28]. The presence of mitochondrial DNA recombination has been proved by some direct evidence [29,30]. Secondly, the tandem duplication and random loss (TDRL) model [31], a commonly accepted hypothetical mechanism to clarify gene rearrangements occurred via tandem duplications of certain genes, followed by random deletion of some gene regions [24,32,33]. Last but not least, the tandem duplication and non-random loss (TDNL) model, which assumes that this process involves complete mitogenome duplication and gene loss. The non-random gene loss depended on their transcriptional polarities and locations in the genome, and resulted in the gene rearrangements with additional non-coding regions [34]. The TDNL model has been applied to explain the gene rearrangements of invertebrate mitogenomes [35,36]. Nevertheless, cases of convergence exist, particularly near hotspots of gene order rearrangements [24], where some flatfishes exhibit particular large-scale tRNA genes rearrangements [37,38].

The Gobioidei belongs to the order Perciformes and comprises about 2210 species [39]. Due to their worldwide distribution from tropical regions to temperate regions [40], these gobioids exhibit prominent variety in morphology, ecology, and behavior among other teleosts [41]. Odontobutidae is one of the basal families within the suborder Gobioidei [42], and this family comprises at least six genera and about 15 species [43]. Thus far, few studies have tackled with gobioid intra-relationships based on morphological and molecular data, especially rarely involving Odontobutids. As a consequence, the biology and classification of gobioids are still controversial [44], despite their evolutionary and ecological importance [45]. For instance, early studies about gobioids regarded the Rhyacichthyidae as the sister to all remainder gobioids [40,42,46,47,48]. However, recent molecular phylogenetic results have indicated that the Rhyacichthyidae + Odontobutidae clade is the sister group of all other gobioid lineages [44,49,50]. As for Odontobutidae, all phylogenetic hypothesis in previous studies just contained few Odontobutidae species and/or partial mitochondrial nucleotide sequences [40,42,44,45,48,49,51,52,53]. In particular, Zang *et al.* [54] analyzed the molecular phylogeny of the family Odontobutidae based on mitochondrial DNA, while they ambiguously explained the method and dataset used for constructing phylogenetic tree, and their intra-relationships of Odontobutidae were different from previous standardized reanalysis of molecular phylogenetic hypotheses (see figures S11 and S12 in [45]). Moreover, recent odontobutid mitogenomic phylogeny did not argue about mitochondrial gene order and mitogenome organization as phylogenetic markers [54]. Therefore, clarifications of the whole taxonomic and evolutionary relationships among Odontobutids remain to be completed.

Considering these perplexities and insufficient above, we report one new mitogenome of *O. yaluensis* and firstly present comparative mitogenomic analyses of *Odontobutis* species in the present study. We compare five *Odontobutis* mitogenomes in detail, regarding mitogenome structure, base composition, codon usage, gene order, evolutionary factors, and the tandem repeats in control regions. The features of this unique gene order and additional intergenic spacers provide sufficient evidence for the TDRL model, accounting for the conserved gene rearrangement in *Odontobutis* mitogenomes. In addition, the phylogenetic trees of the family Odontobutidae are reconstructed based on the concatenated nucleotide sequences of 13 mitochondrial PCGs datasets from seven odontobutids (one for *Micropercops*, one for *Perccottus*, and five for *Odontobutis*). Our comparative analyses of mitogenome sequences and gene rearrangement provide novel insights into the evolutionary relationships within Odontobutidae.

## 2. Results and Discussion

### 2.1. Mitogenome Composition

The new mitogenome of *O. yaluensis* was sequenced, annotated and deposited in the NCBI database (GenBank accession number: KM207149). Aligning overlapping mitochondrial DNA amplifications spanning the whole mitogenome indicated that the total length was 16,988 bp, slightly longer than that of *O. potamophila* (16,932 bp, KF305680) and *O. interrupta* (16,802 bp, KR364945) while significantly shorter than that of *O. sinensis* (17,441 bp, KF154120) and *O. platycephala* (17,588 bp, DQ010651). Moreover, the size of the newly sequenced *O. yaluensis* mitogenome was 79 bp longer than that of another *O. yaluensis* mitogenome (16,909 bp, KM277942), primarily due to the significant sequence variation of intergenic non-coding region between *trnL*(*CUN*) and *trnH*.

**Figure 1:**
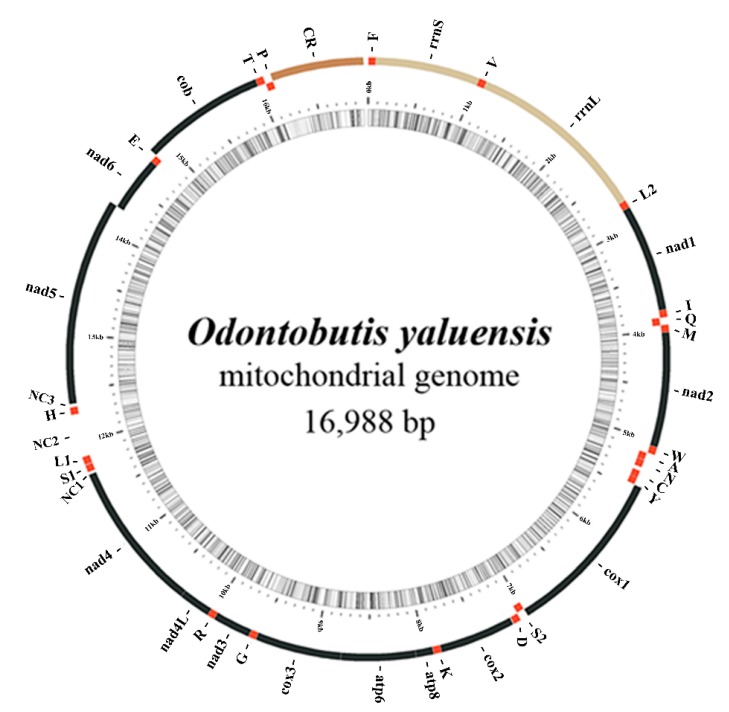
The gene map of *Odontobutis yaluensis* mitogenome. Genes located at the H- or L-strand are mapped outside or inside of the circle. The names of protein-coding genes and rRNAs are expressed by standard abbreviations, while names of tRNAs are abbreviated by a single letter. “CR” refers to the control region, while “NC1”, “NC2” and “NC3” refer to additional large intergenic non-coding spacers. The innermost circle of the images represents (G + C)% per every 5 bp of the mitogenome; the darker lines are, the higher their (G + C)% are. The figure was initially generated by MitoFish and MitoAnnotator program [58], and modified manually.

As expected, it displayed 37 typical mitochondrial genes (13 PCGs, 22 tRNAs, and two rRNAs) and the putative CR. Most gene sequences were on the H-strand, however, eight tRNAs (*trnQ*, *trnA*, *trnN*, *trnC*, *trnY*, *trnS*(*UCN*), *trnE*, and *trnP*) and *nad6* were encoded on the L-strand (Figure 1). The mitogenome structure and individual gene size (Table S1) were largely identical to those of other *Odontobutis* species, with the unusual *trnS-trnL-trnH* gene arrangement which differs from that determined in other non-*Odontobutis* lineages of Gobioidei [53,55,56,57].

Comparative analyses also showed that base composition in five *Odontobutis* species was similar, with a slight A+T bias (Table 1). In addition, the bias against G was prominent at the second and third codon of protein-coding genes, especially at the third codon. Such relaxed selection at the third codon position was considered to result from different natural selection or mutational pressures [59], and might affect the base composition of the whole mitogenomes.

**Table 1:** A+T contents, AT/GC-skew of the mitochondrial genomes of five *Odontobutis* species.

Region	A+T					AT-skew					GC-skew				
	*Osi*	*Opl*	*Oya*	*Oin*	*Opo*	*Osi*	*Opl*	*Oya*	*Oin*	*Opo*	*Osi*	*Opl*	*Oya*	*Oin*	*Opo*
Whole genome	58.91	56.87	55.79	55.33	55.43	0.08	0.09	0.03	0.08	0.02	−0.30	−0.30	−0.31	−0.30	−0.30
Protein-coding genes	58.39	55.86	55.17	54.63	54.76	0.03	0.01	−0.03	−0.01	−0.05	−0.31	−0.32	−0.33	−0.31	−0.32
1st codon position	50.96	48.21	48.43	48.03	48.42	0.07	0.16	0.01	0.14	0.01	−0.05	−0.05	−0.07	−0.05	−0.06
2nd codon position	59.20	58.79	58.57	58.61	58.48	−0.16	−0.38	−0.17	−0.38	−0.17	−0.34	−0.36	−0.35	−0.35	−0.35
3rd codon position	65.00	60.58	58.52	57.27	57.37	0.17	0.26	0.06	0.25	0.03	−0.62	−0.64	−0.63	−0.59	−0.60
tRNA genes	56.63	55.35	55.97	55.11	55.43	0.15	0.14	0.13	0.11	0.11	0.03	−0.15	0.03	−0.14	0.03
*rrnL*	57.85	56.01	54.72	56.30	55.74	0.21	0.24	0.17	0.28	0.19	−0.13	−0.11	−0.11	−0.10	−0.10
*rrnS*	54.95	53.80	53.15	52.00	52.68	0.17	0.26	0.13	0.27	0.12	−0.12	−0.14	−0.14	−0.16	−0.14
Control region	68.77	68.28	64.98	64.79	64.67	0.19	0.01	0.20	−0.01	0.19	−0.25	−0.15	−0.13	−0.13	−0.13

Osi, Opl, Oya, Opo, and Oin indicate *O. sinensis*, *O. platycephala*, *O. yaluensis*, *O. potamophila*, and *O. interrupta*, respectively.

### 2.2. Comparison of Protein-Coding Genes

All PCGs shared ATG start codon, except for *cox1*, which began with GTG. The stop codons varied with TAA, TAG, TA, or T (Table S1), and the incomplete stop codons were presumably completed by post-transcriptional polyadenylation [60]. Comparative analyses showed differences among gobioids that the *cox1* gene stopped with TAG in *O. sinensis* but TAA in other four *Odontobutis* species, and even varied with AGA or AGG in other gobioids [61,62]. The results reveal that the *cox1* gene of gobioids may select a different mechanism for transcription termination during the evolutionary process. Additionally, gene overlapping regions have been detected in all these *Odontobutis* mitogenomes. For example, *atp8-atp6* and *nad4L-nad4* each overlap by seven nucleotides, and *nad5-nad6* share four nucleotides, which agree with those of most other vertebrate mitogenomes [63].

**Figure 2:**
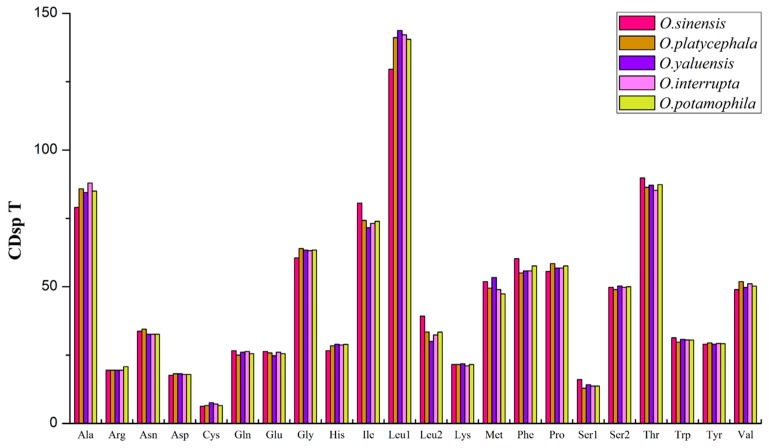
The codon usage pattern of five *Odontobutis* mitogenomes. The codon families are shown on the *X*-axis and CDsp T on the *Y*-axis.

Excluding stop codons, the 13 PCGs in these five *Odontobutis* mitogenomes consisted of 3797–3800 codons (CDs) in total, with a very similar behavior of codon usage (Figure 2). The four most predominant codon families were Leu1 (CUN), Thr, Ala, and Ile, each with more than 70 CDsp T (codons per thousand codons). Among them, Leu1 (CUN), as one of the hydrophobic amino acids, possessed the highest usage bias (129.5–143.7 CDsp T), which might be associated with the encoding function of chondriosome [64]. By contrast, Cys had the least CDsp T.

**Figure 3:**
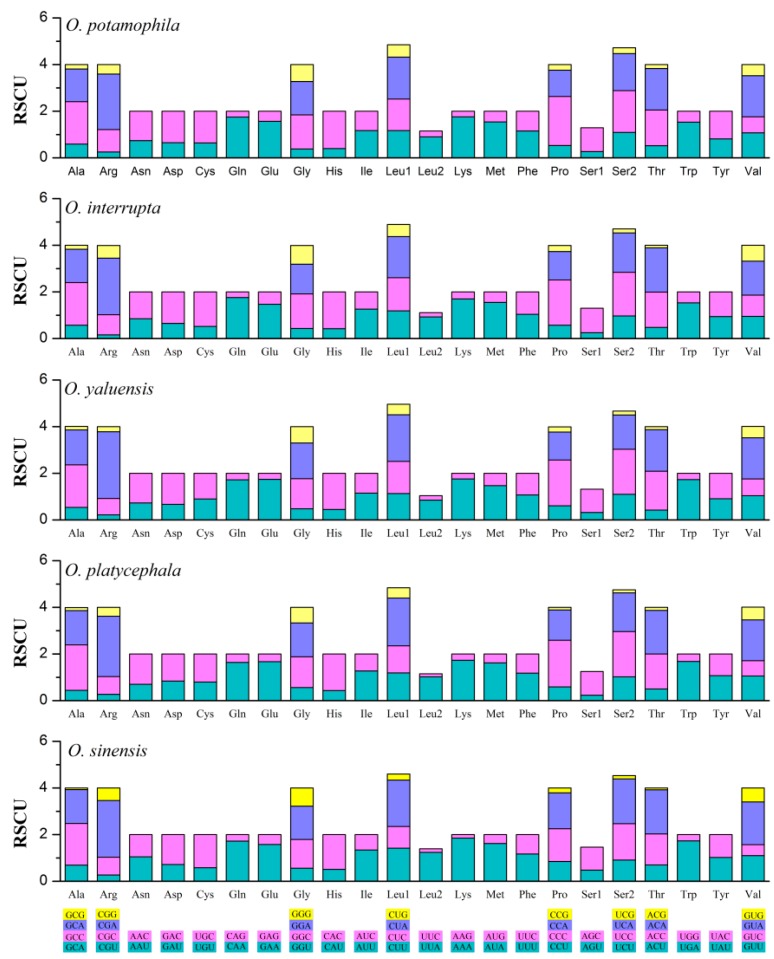
The RSCU of five *Odontobutis* mitogenomes. The codons are shown on the *X*-axis, and the RSCU values are shown on the *Y*-axis.

Subsequently, we utilized the relative synonymous codon usage (RSCU) to determine the preference for particular synonymous codons [65,66]. The codon usage pattern among these five *Odontobutis* species were similar, with both two- and four-fold degenerate codons exhibiting an over-usage of A and T at the third codon positions (Figure 3), which was consistent with other teleosts [67]. This phenomenon might relate to genome bias, optimal selection of tRNA usage, or the efficiency of DNA repair [68,69].

To explore the sequence divergence among *Odontobutis* mitogenomes, we analyzed the pairwise genetic distances based on 13 PCGs (Table 2). The *cox2* gene showed the smallest genetic distance among the 13 single PCG (mean distance: 0.120, Kimura two-parameter distance; K2P), while *nad5* gene showed the largest genetic distance (mean distance: 0.835), revealing different mutation pressures among genes [70]. The K2P pairwise genetic distance of *nad5* gene exhibited low variation within *O. yaluensis*, *O. potamophila*, *O. interrupta*, and *O. platycephala* (averaged 0.165, range 0.062–0.237) but high sequence divergence between these four species and *O. sinensis* (averaged 1.839, range 1.759–1.920). Furthermore, we also calculated the pairwise distance based on amino acid sequences, showing higher values than those calculated by nucleotide sequences. Our results show that synonymous substitutions are less than nonsynonymous substitutions in the PCGs of *Odontobutis* mitogenomes, revealing some protein-coding genes may have experienced positive selection.

**Table 2:** Pairwise genetic distances for 13 PCGs.

Gene	*Osi-Opl*	*Osi-Oya*	*Osi-Opo*	*Osi-Oin*	*Opl-Oya*	*Opl-Opo*	*Opl-Oin*	*Oya-Opo*	*Oya-Oin*	*Opo-Oin*	Mean
*atp6*	0.250	0.228	0.234	0.245	0.214	0.216	0.233	0.097	0.113	0.055	0.189
*atp8*	0.249	0.262	0.288	0.291	0.238	0.266	0.239	0.130	0.138	0.045	0.215
*cox1*	0.148	0.143	0.142	0.153	0.145	0.152	0.146	0.081	0.081	0.040	0.123
*cox2*	0.168	0.157	0.170	0.162	0.123	0.132	0.128	0.066	0.063	0.034	0.120
*cox3*	0.163	0.166	0.187	0.178	0.147	0.172	0.164	0.094	0.106	0.059	0.143
*cob*	0.213	0.208	0.192	0.195	0.176	0.144	0.128	0.093	0.098	0.049	0.150
*nad1*	0.217	0.239	0.248	0.249	0.191	0.192	0.207	0.129	0.132	0.058	0.186
*nad2*	0.228	0.250	0.257	0.258	0.227	0.243	0.247	0.170	0.171	0.063	0.211
*nad3*	0.294	0.279	0.300	0.263	0.232	0.334	0.255	0.207	0.133	0.128	0.243
*nad4*	0.268	0.268	0.267	0.257	0.229	0.246	0.241	0.113	0.118	0.063	0.207
*nad4L*	0.159	0.150	0.207	0.145	0.182	0.272	0.203	0.175	0.095	0.111	0.170
*nad5*	1.920	1.819	1.759	1.860	0.218	0.237	0.229	0.125	0.120	0.062	0.835
*nad6*	0.269	0.278	0.295	0.297	0.271	0.262	0.277	0.126	0.123	0.066	0.226
Nt	0.924	0.934	1.259	1.257	0.292	1.233	1.251	1.125	1.148	0.058	0.948
AA	1.057	1.051	1.519	1.523	0.255	1.483	1.492	1.450	1.462	0.040	1.133

The abbreviations for five scientific names agree with those in Table 1; In addition, “Nt” indicates “the nucleotide of concatenated 13 PCGs”, and “AA” indicates “the amino acid of concatenated 13 PCGs”.

**Figure 4:**
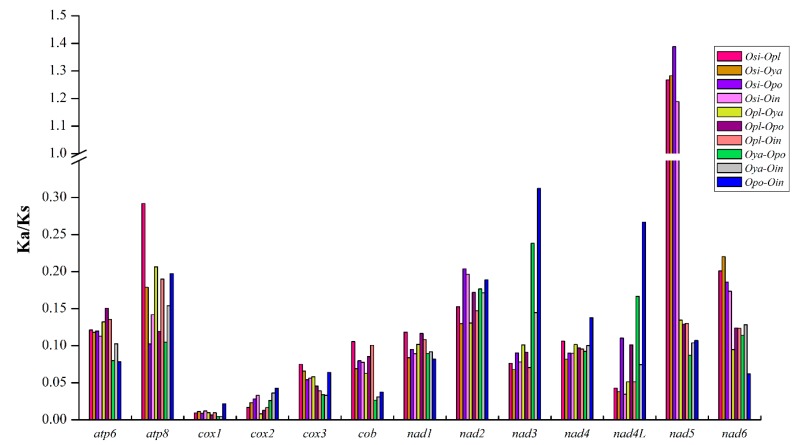
The evolutionary rates (Ka/Ks) for each PCG among five *Odontobutis* mitogenomes. The names of 13 PCGs are shown on the *X*-axis, and the Ka/Ks values are shown on the *Y*-axis.

Among all 10 groups, the Ka/Ks values of most PCGs were less than 0.3 (Figure 4), indicating that they were under purifying selection. However, the Ka/Ks values of *nad5* in these four groups (*Osi-Opl*, *Osi-Oya*, *Osi-Opo*, and *Osi-Oin*) were greater than 1, which showed a strong positive selection. Previous study has illustrated that energetic functional constraints are the major factors shaping different patterns of mitochondrial-encoded protein evolution [71,72]. Combining the Ka/Ks and genetic distance data suggests that *O. sinensis* is a relatively distinct lineage from other *Odontobutis* species, and *nad5* gene has played a crucial role in the evolutionary process of selective adaptation.

### 2.3. High Variations in Control Regions

Previous studies have indicated that the CR of *O. platycephala* contained a tandem repeat (TR) region consisting of 14 copies of 34 bp unit (476 bp in total) [53], and the CR of *O. sinensis* comprised seven 46 bp repeat units (322 bp in total) [57]. However, the CR of *O. potamophila* was composed of non-repetitive sequences [56], which was identical to that of *O. yaluensis* and *O. interrupta*. It was not difficult to find that these TRs bear responsibility for the length heteroplasmy in CRs of *Odontobutis* mitogenomes.

Upstream of CR in most animal mitogenomes, there was a conserved structure including the motif “ATGTA” in TAS-complementary TAS block sequence, which had been suggested as a terminate signal for CR strand synthesis [63]. We had detected the conserved motif “ATGTA” in every repeat unit in the CR of *O. sinensis* and *O. platycephala* (Figures S1 and S2). Buroker *et al.* [15] had proposed the illegitimate elongation model to account for the formation of the repeated sequences in the mitogenome, and this model was targeted at explaining the generation of the TAS motif in the 5′ of the CR. In the present study, the TRs in the *O. sinensis* and *O. platycephala* exactly contained the sequence associated with TAS domain. Thus, the heteroplasmy of TRs in *O. sinensis* and *O. platycephala* mitochondrial CR could be explained by the illegitimate elongation model.

### 2.4. Gene Rearrangement and Possible Mechanisms

For a typical vertebrate mitogenome, the tRNA-gene cluster between *nad4* and *nad5* genes includes *trnH*, *trnS*, and *trnL* genes in this order [63]. However, in the *O. yaluensis* mitogenome, the position of *trnH* gene had been translocated to the downstream of the *trnL* gene in the order S–L–H. This novel gene order was identical to that of recently reported *Odontobutis* mitogenomes with three large intergenic non-coding sequences that respectively named NC1, NC2, and NC3 in this study (Figure 1). Previous study about the mitogenome of *O. platycephala* has reported this novel S–L–H gene order accompanied with three large intergenic spacers [53]. However, due to the lack of comparative mitogenomics data, the authors speculated this phenomenon might have occurred independently in certain species rather than all members of the genus *Odontobutis* [53]. As this gene rearrangement had not been observed in other vertebrates, our results suggest that this gene rearrangement is conserved in *Odontobutis* mitogenomes.

**Figure 5:**
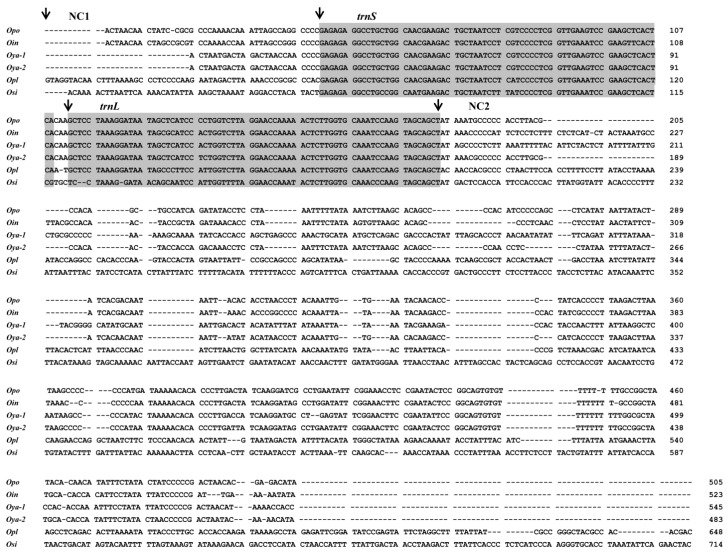

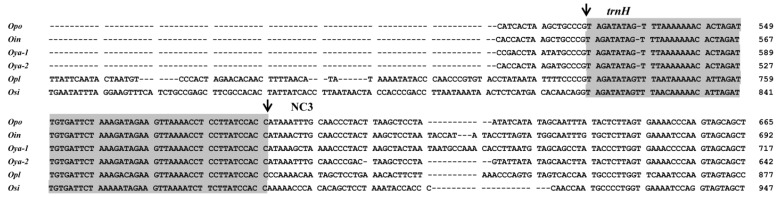
Aligned sequences of gene rearrangement regions of *Odontobutis* mitogenomes. Arrows indicate portions of three intergenic spacers and arranged genes. The positions of black arrows represent the starting sites of different regions. Gray boxes denote the tRNA genes. The numbers reveal the positional relationships among sequences. The abbreviations for their scientific names agree with those in Table 1. In addition, the *Oya-1* indicates the *O. yaluensis* (KM207149), while *Oya-2* indicates another *O. yaluensis* (KM277942).

As shown in Figure 5, these intergenic spacers exhibited high variations among interspecific and intraspecific mitogenomes. The positions and sizes of intergenic spacers in each mitogenome indicate that the NC1 is the pseudogene of *trnH* gene and NC3 appears to be the residual sequence of combined *trnS* and *trnL* genes. Further sequence alignment could also provide evidence for our hypothesis (Figures S3 and S4). In addition, although the sequence similarity between NC2 and CR was low (<50%), we still infer the NC2 as the residual sequence of CR for the following reason: the NC2 in each mitogenome included some residual sequences of conserved sequence blocks (TAS, CSB-C, -D, -F, -1, and -2; Figures S1, S2, and S5–S7).

**Figure 6:**
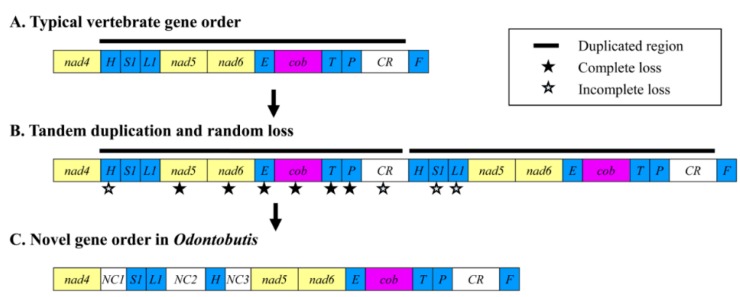
The mechanism proposed for the gene rearrangement in *Odontobutis* mitogenomes in tandem duplication and random loss (TDRL) model. The background colors correspond to the attributes of different gene clusters. The gene names are identical with those in Figure 1.

Considering gene rearrangements occurring by tandem duplication of gene regions and deletions of redundant genes [27,73,74], the present rearranged genes and the associated intergenic spacers (pseudogenes) of *Odontobutis* mitogenomes could be explained by such process as follows (Figure 6): Firstly, tandem duplication occurred in the *trnH-CR* region, and the mitogenome would have contained two sets of the same region (Figure 6B); Then, to maintain the normal function of the mitogenome, one of the duplicated genes and CR randomly lost its function and became a pseudogene or even was completely lost during subsequent evolutionary events. Actually, as described above, NC1, NC2, and NC3 respectively corresponded to *trnH*, CR, and *trnS-L*; however, the approximately 3700 bp sequence (the *nad5-trnP* duplication) left no trace in NC2. This observation supports our hypothesis that the gene rearrangement events have occurred via tandem duplication of the whole *trnH*-CR region. Eventually, the existing gene order and intergenic spacers of the *Odontobutis* mitogenome were established (Figure 6C).

### 2.5. Phylogenetic Relationships

Mitochondrial sequences are widely used to infer phylogenetic relationships among teleosts [5,21,75]. To have a better insight into the phylogenetic interrelationships within Odontobutidae, we obtained the concatenated nucleotide sequences of 13 PCGs from seven Odontobutidae species, including five *Odontobutis* species, one *Perccottus* species and one *Micropercops* species. Besides this, we used *Rhyacichthys aspro* as an outgroup because there was compelling evidence that Rhyacichthyidae was most closely related to Odontobutidae [44,45,49]. The phylogenetic trees reconstructed by two methods (maximum likelihood (ML) and Bayesian inference (BI)) exhibit a coincident topology (Figure 6A). Almost all nodes have high ML bootstrap supports (>90) and Bayesian posterior probabilities (>0.95).

**Figure 7:**
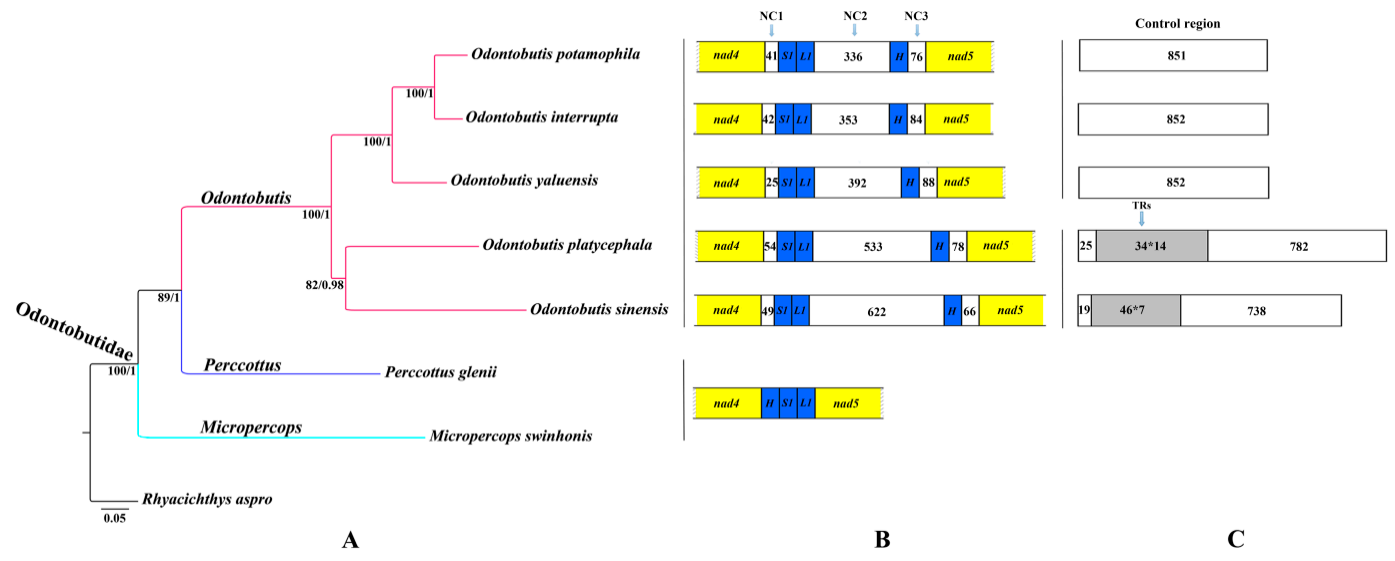
(**A**) Phylogenetic relationships of Odontobutidae species using concatenated nucleotide sequences of 13 mitochondrial PCGs. Numbers at the nodes show ML bootstrap percentages (**left**) and BI posterior probabilities (**right**), respectively; (**B**) Comparison of mitogenome structure between *nad4* and *nad5* genes within Odontobutidae. The protein-coding genes, tRNA genes, and non-coding regions are shown with yellow, blue, and white boxes, respectively. The numbers in white boxes (NC1, NC2, and NC3) represent the number of unassigned nucleotides of intergenic spacers; (**C**) Comparative analyses of the structure of CR among *Odontobutis* species. In white boxes, the numbers indicate the length of fragment. The gray shaded boxes represent the tandem repeat (TR) regions.

Odontobutidae contains six genera (*Odontobutis*, *Perccottus*, *Micropercops*, *Neodontobutis*, *Sineleotris*, *Terateleotris*) [43,76], while no representatives of the latter three genera could be included here or in previous phylogenetic researches. In the present study, the phylogenetic trees showed that five *Odontobutis* species which shared conserved mitochondrial gene rearrangement were clustered into one clade (Figure 7A,B), indicating this gene rearrangement event may occur after *Odontobutis* diverges from other Odontobutidae lineages. In addition, the monophyly of the genus *Odontobutis* was also supported by the sampled taxa (Figure 7A), which was consistent with previous phylogenetic hypotheses [45,52,54]. The phylogenetic topologies exhibited that *O. sinensis* and *O. platycephala* shared a closer relationship, and the remainders formed another distinct clade. In addition, this phylogenetic interrelationship of *Odontobutis* exactly corresponded to the above comparative analysis based on whether they possessed tandem repeats in the mitochondrial control region (Figure 7C). The present study proves that the gene order and genome organization provide useful genetic information for understanding the evolutionary relationships among Odontobutidae species.

## 3. Experimental Section

### 3.1. Samples and DNA Extraction

Specimens of *O. yaluensis* were collected from Dandong in Liaoning Province, China (40°31′22.74′′N, 123°55′14.24′′E), and identified according to Wu *et al.* [77]. Subsequently, caudal fins were preserved in 100% ethanol. Total genomic DNA was extracted by the modified ammonium acetate precipitation protocol [78]. The DNA integrity was examined by electrophoresis in agarose gel, and the purity and concentration were measured on the NanoDrop 2000 (Thermo Scientific, Wilmington, DE, USA).

### 3.2. PCR Amplification and Sequencing

Based on conserved nucleotide sequences of published *Odontobutis* mitogenomes, a total of 20 pairs of species-specific primers (Table S2) were designed using Primer Premier 5.0 software (PREMIER Biosoft International, Palo Alto, CA, USA) for the amplification of the entire *O. yaluensis* mitogenome. To sequence the complete mitogenome and assemble correctly, we make sure that adjacent two fragments overlap more than 50 bp.

The PCR reactions were carried out on an Eppendorf Thermal Cycler (Berlin, Germany) in 25 μL reaction volumes containing 2 μL of each primer, 2 μL PCR buffer II (Mg^2+^), 1.25 mM of dNTPs, 1.25 U LA *Taq* polymerase, about 100 ng template DNA, and sterile doubly-distilled water to final volume. Conditions for PCR amplification were as follows: one initial denaturation step at 94 °C for 2 min; then 94 °C for 30 s (denaturation), 50–58 °C for 45 s (annealing), 72 °C for 1–3 min (extension) for 35 cycles; followed by a final extension step at 72 °C for 10 min. The PCR products were examined by 1.0% agarose gel electrophoresis, and purified using the TaKaRa MiniBEST Agarose Gel DNA Extraction Kit (Takara, Dalian, China). Sequencing was completed in Tsingke Biotech Co., Ltd. (Wuhan, China).

### 3.3. Gene Annotation and Sequence Analysis

The DNA sequences were assembled using SeqMan program of Lasergene 7.0 (DNAstar, Madison, WI, USA) to create complete mitogenome. During the walking sequencing of large fragments, we regularly examine the assembly to ensure its reliability. The annotation of 13 PCGs and two rRNAs, and the definition of each gene boundaries were determined by both DOGMA [79] and MitoFish [58] programs. tRNAs and their secondary structures were predicted by tRNAscan-SE 1.21 [80], and the cut-off value was 1. Non-coding regions were identified via sequence homology with Clustal W2 [81]. Tandem repetitive elements were detected by using the Tandem Repeats Finder 4.04 [82].

Nucleotide base compositions and codon usage were calculated with MEGA 5.2 [83]. AT-skew ((A−T)/(A+T)) and GC-skew ((G−C)/(G+C)) were used to measure nucleotide bias [84]. The genetic distance of different PCGs were also analyzed in MEGA 5.2 [83]. The Ks and Ka in each protein-coding gene were determined by DnaSP 5.0 [85] for ten groups: *O. sinensis-O. platycephala* (*Osi-Opl*), *O. sinensis-O. yaluensis* (*Osi-Oya*), *O. sinensis-O. potamophila* (*Osi-Opo*), *O. sinensis-O. interrupta* (*Osi-Oin*), *O. platycephala-O. yaluensis* (*Opl-Oya*), *O. platycephala-O. potamophila* (*Opl-Opo*), *O. platycephala-O. interrupta* (*Opl-Oin*), *O. yaluensis-O. potamophila* (*Oya-Opo*), *O. yaluensis-O. interrupta* (*Oya-Oin*), *O. potamophila-O. interrupta* (*Opo-Oin*). The gene map of the *O. yaluensis* mitogenome was generated by MitoFish and MitoAnnotator program [58].

### 3.4. Phylogenetic Analyses

A total of seven Odontobutidae species were used to reconstruct the phylogenetic trees. Beside this, we selected *Rhyacichthys aspro* (AP004454) as an outgroup (Table S3). The nucleotide sequences of 13 mitochondrial PCGs were concatenated and a multiple sequence alignment was performed with Clustal W built-in MEGA 5.2 [83]. Phylogenetic analyses were carried out by both ML and BI methods. We implemented the ML analyses in RAxML version 8.0.0 (BlackBox webserver; http://embnet.vital-it.ch/raxml-bb/) to generate phylogenetic trees under GTR+G+I model [86]. The Bayesian analyses was performed using Mrbayes 3.2.4 [87] with four independent chains running for 3 million generations, sampling a tree every 1000 generations, the first 750 trees were removed as burn-in and the remaining trees were used to calculated Bayesian posterior probabilities (BPP). Phylogenetic trees were viewed and edited in Figtree 1.4.0 [88].

## 4. Conclusions

The mitogenome of *O. yaluensis* is similar to those of other four *Odontobutis* species. The identical gene rearrangement of *trnS-trnL-trnH* tRNA cluster observed in these mitogenomes suggests that this unique gene order is conserved within the genus *Odontobutis*. The present rearranged genes and associated intergenic spacers reveal that this gene rearrangement results from tandem duplication and random loss (TDRL) of large-scale gene regions. Phylogenetic analyses of the family Odontobutidae support *Odontobutis* species which share gene rearrangement forming a monophyletic group, and the interspecific evolutionary relationships within the genus *Odontobutis* are consistent with the features, whether or not they share tandem repeats in their control regions.

## Supplementary Materials

Supplementary materials can be found at http://www.mdpi.com/1422-0067/16/10/25031/s1.
