# Supplementary material for: Comparative Mitogenomics of the Genus Odontobutis (Perciformes: Gobioidei: Odontobutidae) Revealed Conserved Gene Rearrangement and High Sequence Variations
Source: Int J Mol Sci. 2015 Oct 20;16(10):25031–49. doi: 10.3390/ijms161025031 (PMC4632788; doi:10.3390/ijms161025031)
Supplement: Supplementary file 1 [file ijms-16-25031-s001.pdf]

Supplementary Information

Table S1. Characteristics of the mitochondrial genes of *Odontobutis yaluensis*.

Gene Name	Position		Size (bp)	Codon		Strand	Intergenic Nucleotide (bp)
	From	To		Start	Stop		
tRNA-Phe	1	69	69	–	–	H	0
12S rRNA	70	1021	952	–	–	H	0
tRNA-Val	1022	1093	72	–	–	H	0
16S rRNA	1094	2756	1663	–	–	H	0
tRNA-Leu	2757	2829	73	–	–	H	0
ND1	2830	3806	977	ATG	TA-	H	0
tRNA-Ile	3807	3876	70	–	–	H	−1
tRNA-Gln	3876	3946	71	–	–	L	−1
tRNA-Met	3946	4014	69	–	–	H	0
ND2	4015	5060	1046	ATG	TA-	H	0
tRNA-Trp	5061	5133	73	–	–	H	5
tRNA-Ala	5139	5207	69	–	–	L	1
tRNA-Asn	5209	5281	73	–	–	L	34
tRNA-Cys	5316	5382	67	–	–	L	1
tRNA-Tyr	5384	5451	68	–	–	L	1
CO I	5453	7006	1554	GTG	TAA	H	0
tRNA-ser	7007	7077	71	–	–	L	4
tRNA-Asp	7082	7152	71	–	–	H	5
CO II	7158	7848	691	ATG	T--	H	0
tRNA-Lys	7849	7922	74	–	–	H	1
ATPase8	7924	8088	165	ATG	TAA	H	−7
ATPase6	8082	8764	683	ATG	TA-	H	0
CO III	8765	9549	785	ATG	TA-	H	0
tRNA-Gly	9550	9620	71	–	–	H	0
ND3	9621	9966	346	ATG	T--	H	0
tRNA-Arg	9967	10,035	69			H	0
ND4L	10,036	10,332	297	ATG	TAA	H	−7
ND4	10,326	11,708	1383	ATG	T--	H	25
tRNA-Ser	11,734	11,801	68	–	–	H	3
tRNA-Leu	11,805	11,877	73	–	–	H	392
tRNA-His	12,270	12,337	68	–	–	H	88
ND5	12,426	14,261	1836	ATG	TAA	H	−4
ND6	14,258	14,779	522	ATG	TAA	L	0
tRNA-Glu	14,780	14,847	68	–	–	L	5
Cyt b	14,853	15,993	1141	ATG	T--	H	0
tRNA-Thr	15,994	16,065	72	–	–	H	2
tRNA-Pro	16,068	16,137	70	–	–	L	0
Control region	16,138	16,988	851	–	–	H	0

Negative numbers indicate overlapping nucleotides; T-- and TA- represent incomplete stop codons; Numbers correspond to the intergenic nucleotides separating adjacent genes.

Table S2. Primers designed for amplifying mitochondrial genome of *Odontobutis yaluensis*.

Forward	Sequence (5′-3′)	Reverse	Sequence (5′-3′)
OY1F	TTAGCCCACGACACCTTG	OY1R	TGTTACGACTTGCCTCCC
OY2F	AGGACTTGGCGGTGCTTTA	OY2R	CGGGTTTGCCCTTTGTTAG
OY3F	AAAAAGACACACGGAGCAA	OY3R	AACCAAAGATGGGGATAGG
OY4F	TATGAATGGCATCACGAGG	OY4R	CTGGAGAAGTCCGCAAGG
OY5F	CGGAGAAATCCAGGTCAGTT	OY5R	ATTCAGGCGAGGAGTCAGTG
OY6F	TGCTTCCACTACACCACTTC	OY6R	GGAGAGCGAGGATGATTTA
OY7F	CCTATCCACCTGACAAAAAC	OY7R	GAGTAGAAAAGATGGGGGG
OY8F	ACCAGCGAGCATCAATCTAC	OY8R	GCAAGGTGGCTAATCAACTA
OY9F	CCTCTTCTGATTCTTCGGAC	OY9R	TGGGATTACTATTCGGTGGT
OY10F	CAAGGCAGGGTTGTGGGTT	OY10R	TTGGGTGGTGGGTGTGAAG
OY11F	AACAGAAGGGCACAAATGAG	OY11R	TAAGGGTGAGGGAGTGGATG
OY12F	CCAAGGACATCACACACCC	OY12R	GCTGAGAATGCGAGAAGGA
OY13F	TACGGCTCCACCTTCTTTGT	OY13R	TAGGTTCCAGCATTTAGGCG
OY14F	AGCCAGCCAAAAACACATA	OY14R	GCTTGCCATAAACCATCAG
OY15F	CTGGGATGATCTGTCTACGAC	OY15R	GGGACGAAGATTGTTGAGTAG
OY16F	TTTGTAAGTCTCCTGCCCC	OY16R	CCCAGTAGTGATGCTTCCTC
OY17F	TGGCATACCCTTTTTAGCAG	OY17R	GGAGGATTAGTGGCAGTTGT
OY18F	TCCCCGAAAGACAGAAAACT	OY18R	GATGGCGTAGGCAAATAAGA
OY19F	AGGCTTCTCCGTAGACAAC	OY19R	CCCTGAAATAGGAACCAAA
OY20F	ATCTGCACTAGTAGCTCAACG	OY20R	GATAGTAAAGTCAGGACCAAGC

Table S3. Taxonomy, GenBank accession number, and mitogenome size of species used in the phylogenetic analyses.

Family	Genus	Species	Accession Number	Size (bp)
Odontobutidae	*Odontobutis*	*Odontobutis potamophila*	KF305680	16,932
		Odontobutis interrupta	KR364945	16,802
		Odontobutis yaluensis	KM207149	16,988
		Odontobutis platycephala	DQ010651	17,588
		Odontobutis sinensis	KF154120	17,441
	Perccottus	*Perccottus glenii*	KC292213	16,487
	Micropercops	*Micropercops swinhonis*	KF040334	16,493
Rhyacichthyidae	*Rhyacichthys*	*Rhyacichthys aspro*	AP004454	16,518

```
                                TRs
O.sin-NC2   ---------ATGACTC---------------------CACCATTCCAC--------CCACTTATG-----------GTATTA-CACC----------------CCTTTATTAA------- 47
O.sin-CR    TTTACATATATGTTTCATTAAACATACTATGTATTATCACCATTTCTCGAATTTAACCATTCATTAAACATACTATGTATTATCACCATTTCTCGAATTTAACCATTCATTAAACATACT 120
                     ***  **                     ******* * *        *** * **            ****** ****                * ** *****

O.sin-NC2   -TTTACTATCCTCATACTT---ATTTATCTTTTTA-----CATATTTTTTAC-----CCAGTC-----ATTT---CACTGATTAAA-ACACCACCCGTGACTGCC-CTTCTC----CTTA 139
O.sin-CR    ATGTATTATCACCATTTCTCGAATTTAACCATTCATTAAACATACTATGTATTATCACCATTTCTCGAATTTAACCATTCATTAAACATACTATGTATTATCACCATTTCTCGAATTTAA 240
             * ** ****  ***   *   ***** *  ** *     **** * * **      ****      ****    ** * ****** * ** *    * *   **  *****     * *

O.sin-NC2   CCCTACCTCTTACATACAA---ATTCTTAC-AT------AAAGTAGCAA------AAACA-----------ATTACCAATAGTTGAATCTGAATATACATA-ACCAACTTTGATATGGG- 230
O.sin-CR    CCATTCATTAAACATACTATGTATTATCACCATTTCTCGAATTTAACCATTCATTAAACATACTATGTATTATCACCATTTCTCGAATTTAACCATTCATTCATCAACAGTAAATTAAGG 360
            ** * * *   ****** *   *** * ** **      **  ** * *      *****           ** **** *  * **** * *  ** ***  * ****  * *  *  *
                TAS
O.sin-NC2   -----------------AATTAACC-----------TAACATTTAGC---CACTACTCAGCAGCCT---CCAC----------CGTAACA---ATCCTG--------TGTATACTTTGAT 295
O.sin-CR    TATACATATATACAAACAATTAACTATCAACATCTATAGAATTTAATGTATATTACCCAAATATTTACCCCACGAAATTAAGACCTAACATAAATTTTAATAAAACATATATACCAGGAT 480
                             *******            **  *****      * *** **      *   ****          * *****   **  *         * *****   ***

O.sin-NC2   TTA--------------TTACAA----------------AAAAC--TTACCTCAA-CTTGCTAATACC--TACTTAAAT-TCAAGCACAA--ACCATAAA--CCCTATTTAA--ACCTTC 373
O.sin-CR    TCAAGATCTAGTCAAGATAACAAGACTATGCAGATAAGGAAGACATTTAAGTCAAGCATAGTAAATTCGGTTCTTGAAGGTGAGGGACAATAATCGTGGGGGTTTCACTTAGTGAATTAT 600
            * *              * ****                ** **  ***  **** * * ***    *  * *** **  * * * ****  * * *         * ***   *  *
                    CSB-D
O.sin-NC2   TCCT----------TACTGT---------ATTTATTA-------TCACCAT-----------AACTGACATAGTACAATTTTTAGTAAAGTATAAAGAAC-----AGACCTCCATACTAA 451
O.sin-CR    TCCTGGCATTTGGTTCCTATTTCAGGGCCATTTATTGATATTATTCCCCATTCTTTCCTTAAAACGGGCATA----AGTTGTTGGTGGAGTATATACTCCTCGTTACACCAC-ATGCCGA 715
            ****          * ** *         *******        ** ****           *** * ****    * ** ** **  ****** *   *     * *** * ** *  *

O.sin-NC2   CCATTTTT----ATTGACTAACCTAAGACTTTAT-TCACCCTCTCATCC---------------------------------CAAGGG-------------TGCACCTAAATAT------ 514
O.sin-CR    GCGTTCTTTCTAATGGACTAGGTTATTTTTTTTTGTCCTCCTTTCATCTGGCATTTCAGAGTGCAGCGCTAAGACTTGTTGACAAGGGAGATCATTTTTCTTGCATGCAAGTATAATATT 835
             * ** **    ** *****   **    *** * **  *** *****                                  ******             ****   ** ***
                CSB-1                                   CSB-C                                                      CSB-F
O.sin-NC2   TCAGAACT--ACTGAATATTTAGGA--AGTTTCAT--CTG---CCGAG----------------CTTCGCC--ACAC--------TATTATCACCTTAA------------TAA------ 581
O.sin-CR    TTAATGTTGAAATGACTTTTTAAGATAAATTGCATAACTGATATCAAGGGCATAATGATAATTATTTCTCCTAACATATCTGATATATTCCCCCCTTTGCTTTTTTCCGCGTAAACCCCC 955
            * *    *  * *** * **** **  * ** ***  ***    * **                 *** **  ***         ****  * ****              ***
               CSB-2
O.sin-NC2   -CTACCACCCGACC-TTAATAAATAACTCTCAT-----------------GACACAACAG------------------------------------------------------------
O.sin-CR    CCTACCCCCCCATTACTCGTAAGTTATTATTATTCCTGAAAACCCCCCCGGAAACAGGAGAACCTCGAGTTGGGTATTTAATTGTTCTAATATGCATTATTTATAATATTAAAATAATTT 1075
             ***** *** *    *  *** * * * * **                 ** ***  **

O.sin-NC2   ----
O.sin-CR    ATTT  1079
```

Figure S1. Sequence alignment between NC2 and CR from *Odontobutis sinensis* mitogenome. The red sequence in the black box represents the one unit of tandem repeats in the control region of *O. sinensis* mitogenome; the conserved "ATGTA" motif is highlighted with green background; the conserved sequence blocks are highlighted with different marks. The numbers on the right of sequences reveal the positional relationships among sequences. The "*" means the corresponding two bases are same.

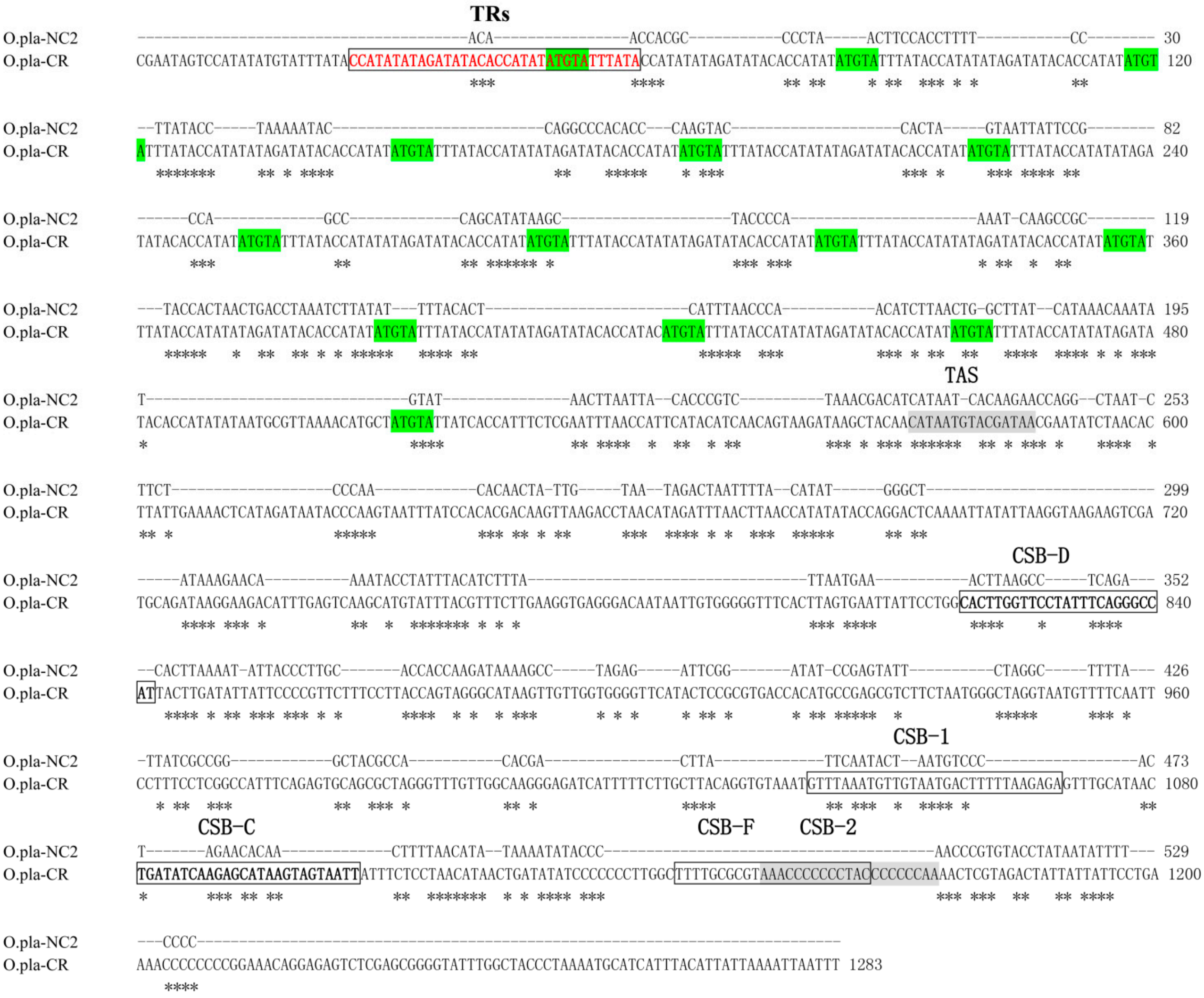

Figure S2. Sequence alignment between NC2 and CR from *O. platycephala* mitogenome. The red sequence in the black box represents the one unit of tandem repeats in the control region of *O. sinensis* mitogenome; the conserved "ATGTA" motif is highlighted with green background; those conserved sequence blocks are highlighted with different marks. The numbers on the right of sequences reveal the positional relationships among sequences. The "*" means the corresponding two bases are same.

```
O.pot-NC1      --------------ACTAACAACTA---TCCGCGCCCAAA---ACAAATTAG-----CCAGG-CCCC- 41
O.pot-trnH     GTAGATATAGTTTAAAAAAACACTAGATTGTGATTCTAAAGATAGAAGTTAAAACCTCCTTATCCACC 68
                             *.:**..****   *  *.   * ***   * **.***.     **: .  **.*

O.int-NC1      ----------ACTAACAA----CTAG-CCGCG--TCCAAAACCAAA--TTAG-----CCGGGCCCC-- 42
O.int-trnH     GTAGATATAGTTTAAAAAAACACTAGATTGTGATTCTAAAGATAGAAGTTAAAACCTCCTTATCCACC 68
                           *** **    ****   * *  ** ***   * *  ***      **    **

O.yal-NC1      --------------ACTAATGACTAGACT----------------------AACC-----AACCCC- 25
O.yal-trnH     GTAGATATAGTTTAAAAAAACACTAGATTGTGATTCTAAAGATAGAAGTTAAAACCTCCTTATCCACC 68
                              *.:**: ****** *                       ****     *:**.*

O.pla-NC1      GTAGGTACAACTT--TAAAAGCCCTC----------CCCAAGAATAGACTTAAAACCCGCG--CCCAC- 54
O.pla-trnH     GTAGATATAGTTTAATAAAAACATTAGATTGTGATTCTAAAGACAGAAGTTAAAACCTCCTTATCCACC 69
               ****.** *.  **  *****.*.  *.          * .****.:..* ********   *   ****

O.sin-NC1      ----ACAAAACTTAATTCAAAACAT---ATT--------AAAGCTAAAA--TAGGACCTACATACT---- 49
O.sin-trnH     GTAGATATAGTTTAACA-AAAACATTAGATTGTGATTCTAAAAATAGAAGTTAAAATCTTCTTATCCACC 69
                   * * *  ****   *******   ***         ***  ** **  **  * ** * **
```

Figure S3. Sequence alignment between NC1 and trnH from five *Odontobutis* mitogenomes. The “*” means the corresponding two bases are same, the “:” means the corresponding two bases are matched, and the “.” means the corresponding two bases are mismatched. The numbers on the right of sequences reveal the positional relationships among sequences.

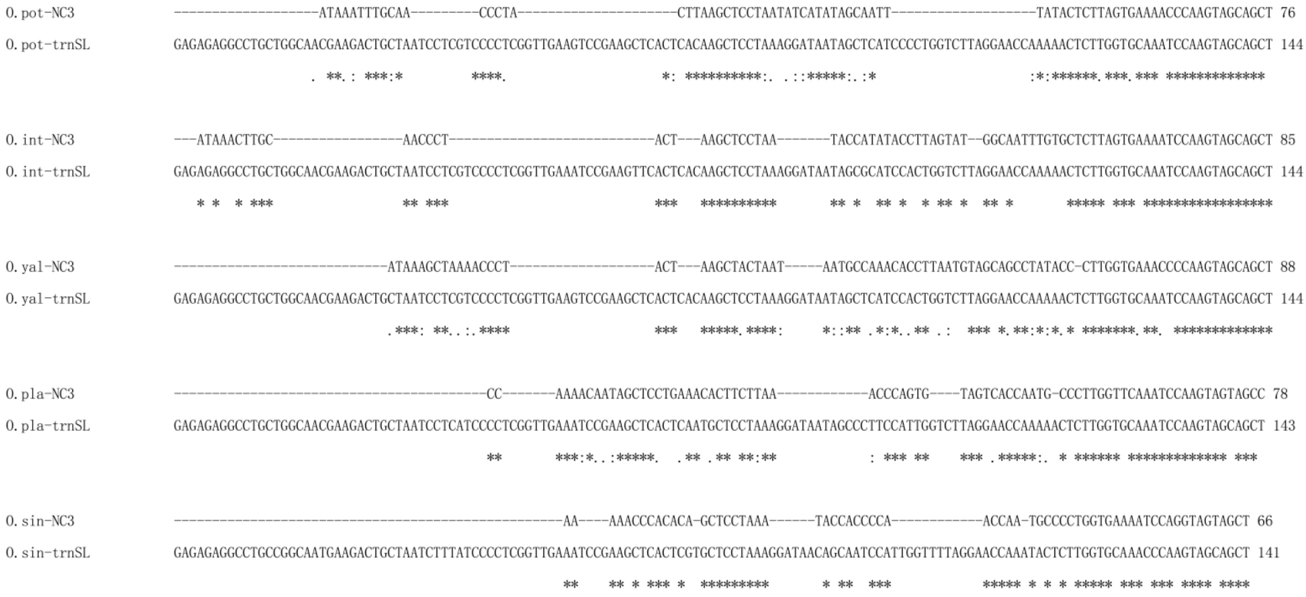

Figure S4. Sequence alignment between NC3 and trnS-L from five *Odontobutis* mitogenomes. The "*" means the corresponding two bases are same, the ":" means the corresponding two bases are matched, and the "." means the corresponding two bases are mismatched. The numbers on the right of sequences reveal the positional relationships among sequences.

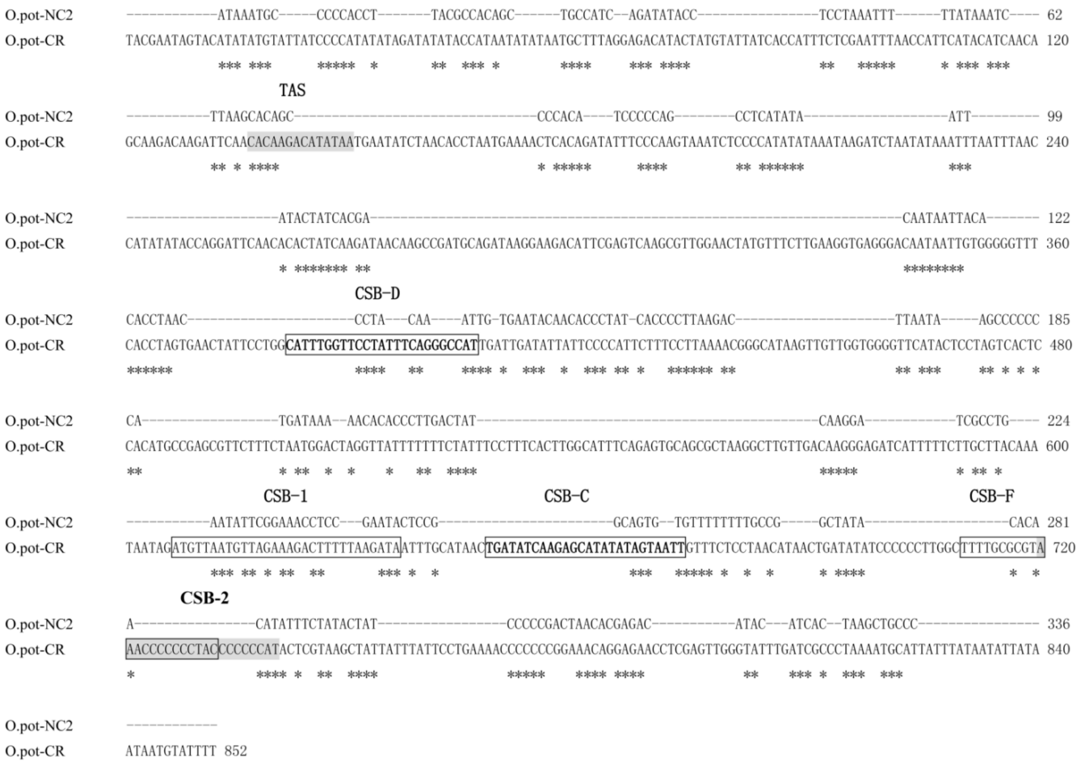

Figure S5. Sequence alignment between NC2 and CR from *O. potamophila* mitogenome. Those conserved sequence blocks are highlighted with different marks. The numbers on the right of sequences reveal the positional relationships among sequences. The "*" means the corresponding two bases are same.

```
O.int-NC2     ----------ATAAACCCCCATTCTCCTCTTTCTCT--CATCTACTA--------AATGCCTTA--------------------CGCCA-----------------------CAACTACC 57
O.int-CR      TACGAATAGTACATATATGTATTATCCCCATATATAGATATATACCATAATATATAATGCTTTAGGAGACATACTATGTATTATCACCATTTCTCGAATTTAACCATTCATACATCAACA 120
                        * * *     *** *** * *        ** *** *        ********                    * ***                       ** * **
                                  TAS
O.int-NC2     GCTAGATAA------ACAC----CCTA-----AATTTCTA------------------TAAGTGTT----AAGCACAGCCCCTCA------------ACCTCCTATAACT---------- 118
O.int-CR      GCAAGATAAGATTCAACACAAGACATATAATGAATATCTAACACCTAATGAAAACTCACAGATATTTCCCAAGTAAATCTCCCCATATATAAATAAGACCTAACATAAATTTAATTTAAC 240
              ** ******      ****    * **     *** ****                   *  * **    *** * * * ** **            ****   **** *

O.int-NC2     --------------------ATTCTATCACGACAATAATT---------------AAACACCCG-------------------GCCCC-------------ACAA--ATTAT-------- 161
O.int-CR      CATATATACCAGGATTCAACATACTATCAAGATAACAAGTCGATGCAGATAAGGAAGACATTCGAGTCAAGCGTTGGAACTATGTTTCTTGAAGGTGAGGGACAATAATTGTGGGGGTTT 360
                                  ** ****** ** ** ** *               * ***  **                   *   *             ****  *** *
                                         CSB-D
O.int-NC2     ---------AAATA-----------------------CAAGACCCC--------ACTATCGCCC--------CTTAAGAC------------TTAATAAACCCC-----CCCAATAAAAA 216
O.int-CR      CACCTAGTGAACTATTCCTGGCATTTGGTTCCTATTTCAGGGCCATTAATTGATATTATTCCCCATTCTTTCCTTAAAACGGGCATAAGTTGTTGGTGGAGTTCATACTCCTAGTCACTC 480
                       ** **                       ** * **          * ***  ***        ***** **            **  *  *   *     ** * * *

O.int-NC2     CACA--------------------------------------------CCCTTGACT----AT----------------------------CAAGGATAGCC------------------ 242
O.int-CR      CACATGCCGAGCGTTCTTTCTAATGGACTAGGTTATTTTTTTCTATTTCCTTTCACTTGGCATTTCAGAGTGCAGCGCTAAGGCTTGTTGACAAGGGAGATCATTTTTCTTGCTTACAAA 600
              ****                                            ** ** ***    **                            *****     *
                              CSB-1                                    CSB-C                                                 CSB-F
O.int-NC2     ---TGGATATT-----CGGAAA--CTTCC-------GAAT------ACT----CCGGCAGTGTGT-------TTTTTTTGCC-------------------------GGCTAT-GCACA- 301
O.int-CR      TAATAGATGTTAATGTTGGAAAGACTTTTTAAGATAAATTGCATAACTGATATCAAGAGCATATATAGTAATTGTTTCTCCTAACATAACTGATATATCCCCCCTTGGCTTTTGCGCGT 720
                 * *** **      ***** ***           ***      ***     *   **  * *       ** ***  **                         **** * ** *
                      CSB-2
O.int-NC2     -----------------CCACATTCCTA---TATTAT--------------CCCCCGA-------------TTGA--------------------AAAATA-------TACACCACTAAG 347
O.int-CR      AAACCCCCCCTACCCCCCCATACTCGTAAGCTATTATTTATTCCTGAAACCCCCCCGGAAACAGGAGAACCTCGAGTTGGGTATTTGGTCGCCCTAAAATGCATTATTTATAATATTAAA 840
                               *** * ** **   ******              ******              * **                    *****        ** *  * ***

O.int-NC2     CTGCCC------ 353
O.int-CR      ATAATGTATTTT 852
               *
```

Figure S6. Sequence alignment between NC2 and CR from *O. interrupta* mitogenome. Those conserved sequence blocks are highlighted with different marks. The numbers on the right of sequences reveal the positional relationships among sequences. The "*" means the corresponding two bases are same.

```
                                   TAS
O.yal-NC2   -----ATAGCCCCTCT------------------TAAAT--------TTTTACATTCTACTCTA-------TTTTATTTGCTG-CGCC----CCCAAA---AAGCA---AAATATCACCA 71
O.yal-CR    TACGAATAGTACATATATGTATTATCACCATATATAGATATGCACCATTATATATAATGCTTTAGGAGACATACTATGTATTATCACCATTTCTCGAATTTAACCATTCATACATCAACA 120
                 ****  * * *                  ** **        ** ** **  * ** **       *  *** *  *   * **   * * **   ** **   * * **** **

O.yal-NC2   CCA-GCTGAG------CCCAAACTGCATAATG----------------------CTCA-----------------GACGACCCACTATTTAGC---ACCCTAACA---------------- 127
O.yal-CR    GTAAGATAAGATACAACACAAGACATATAATGAATATTTAACGCCTTATGAAAACTCATAGATATAACCCAAGTAAATAACCTCATATCAAGTTAAGACCTAACATAAACTTAATTGAAT 240
              * * * **      * ***     ******                      ****                  *  ***   ***  **      *******

O.yal-NC2   -ATATATTTCAG--------ATATT------------------TATAAATACGGGGCATATGCAA--TAA---TTGACACTACATATTT---ATATAA---ATTATAAATACGAAAG--- 206
O.yal-CR    CATATATACCAGGATTCAAAATACTATCAAGATAACAAGCCGATGCAGATAAGGAAGACATTCGAGTCAAGCGTTGGAACTACGTTTCTTGAAGGTGAGGGACAATAATTGTGGGGGTTT 360
             ******  ***        *** *                  *  *** **   * ** * *   **   ***  ***** * * *   *  * *   *  **** *  *   *

                                 CSB-D
O.yal-NC2   ----------ACCACTACCAACTTT--------ATT--AAGGC------TCAATA--AGCCCCCATACTA-----AAAAC------------------------ACACCCTTGACCATCA 269
O.yal-CR    CACCTAGTGAACTATTCCTGGCATTTGGTTCCTATTTCAGGGCCATTAATTGATATTATTCCCCATTCTTTCCTTAAAACGGGCATAAGTTGTTGGTGGAGTTCATACTCCTAGTCACTC 480
                      **  * * *  * **        ***  * ***      *  ***  *  ****** **      *****                        * ** * *   **

O.yal-NC2   AGGATGCCTGAGTATTC--------GGA-----------------ACTTCCG-------AATATTCCGG----CAGTG--------TGTT--------------TTTTTTTG-------- 324
O.yal-CR    CACATGCC-GAGCGTTCTTTCTAATGGACTAGGTTATTTTTTTCTATTTCCTTTCACTTGGCATTTCAGAGTGCAGCGCTAAGACTTGTTGACAAGGGAGATCATTTTTCTTGCTTACAA 599
               ***** ***  ***        ***                 * ****           *** * *    *** *        ****              ***** ***

                    CSB-1                            CSB-C                                                 CSB-F
O.yal-NC2   ----------------------------------------GCGCTACC-ACACCAA-----------------ATTTCC--------------TATAT---------------------- 350
O.yal-CR    ATAATAAATGTTAATGTAAAAAGACTTTTTAAGATGATTTGCATAACTGATATCAAGAGCATATATAGTAATTATTTCTCCTAACATAACTGATATATCCCCCCTTGGCTTTTGCGCGTA 719
                                                    **   **  * * ***                 *****               *****

               CSB-2
O.yal-NC2   ---------TATCCCCGA-----------CTAACAT---------AAAACCACCCCGA----------------------------------CCTAATATGCCC---------------- 392
O.yal-CR    AACCCCCCCTACCCCCCCATACTCGTAAGCTATTATTTATTCCTGAAAACCCCCCCGGAAACAGGAGAACCTCGAGTTGGGTCTTTGATCACCCCAAAATGCGTTATTTATAATATTAAA 839
                     ** ***** *          ***  **         ****** *****                                   ** ** ****

O.yal-NC2   ------------
O.yal-CR    ATAATGTATTTT 851
```

Figure S7. Sequence alignment between NC2 and CR from *O. yaluensis* mitogenome. Those conserved sequence blocks are highlighted with different marks. The numbers on the right of sequences reveal the positional relationships among sequences. The "*" means the corresponding two bases are same.
